# Challenges in Differentiating Uterine Mesenchymal Tumors—Key Diagnostic Criteria

**DOI:** 10.3390/jcm14134644

**Published:** 2025-07-01

**Authors:** Karolina Daniłowska, Małgorzata Satora, Krzysztof Kułak, Anna Kułak, Rafał Tarkowski

**Affiliations:** 1 Student’s Scientific Association, I Chair and Department of Gynecological Oncology and Gynecology, Medical University of Lublin, Staszica 16 Str., 20-081 Lublin, Poland; msatoraa@gmail.com; 2 I Chair and Department of Gynecological Oncology and Gynecology, Medical University of Lublin, Staszica 16 Str., 20-081 Lublin, Poland; krzysztof.kulak@gmail.com (K.K.); rafaltar@yahoo.com (R.T.); 3 Department of Diagnostic and Microsurgery of Glaucoma, Medical University of Lublin, Chmielna 1 Str., 20-079 Lublin, Poland; puzioan@gmail.com

**Keywords:** leiomyomas, sarcomas, magnetic resonance imaging, positron emission tomography, ultrasound, endometrial stromal sarcomas

## Abstract

**Background**: Uterine fibroids are the most common tumors in gynecology, detected in up to 80% of patients at various points in their lives. Uterine sarcomas account for 3% to 7% of all uterine cancers. The diagnosis of uterine fibroids is possible through ultrasonography (US), but this method has many limitations. More accurate examinations include magnetic resonance imaging (MRI) and positron emission tomography (PET) scans. **Methods**: This study evaluates MRI and PET in differentiating uterine fibroids from sarcomas. MRI uses T2-weighted and diffusion-weighted imaging (DWI), while PET assesses metabolism and estrogen receptor activity using [18F] fluorodeoxyglucose (FDG) and 16α-[18F]-fluoro-17β-estradiol (FES). **Results**: MRI allows for the identification of uterine fibroids when they exhibit good delineation and low intensity in T2-weighted images and DWI. Uterine sarcoma is characterized by moderate to high signal intensity on T2-weighted imaging, irregular borders, high signal intensity at high DWI values, and a decreased apparent diffusion coefficient. PET imaging with FDG and FES is a useful tool in differentiating uterine fibroids from sarcomas. Uterine sarcomas exhibit greater FDG uptake than smooth muscle fibroids, although cases of similar uptake do occur. On the other hand, FES provides information about estrogen receptors (ERs). **Conclusions**: Future research should focus on conducting standardized imaging studies, which would facilitate the inclusion of larger patient cohorts. This, in turn, would enable the development of specific diagnostic guidelines, ultimately leading to more accurate diagnoses and reducing the difficulty of differentiating these tumors through imaging.

## 1. Introduction

Uterine leiomyoma (LMM) is the most common tumor in gynecology, detected in up to 80% of patients at various points in their lives [1]. Uterine fibroids are often diagnosed with ultrasound (US) examination; however, this technique has limitations, related to the precise characterization of solid masses in the uterine muscle [2]. In magnetic resonance imaging (MRI) examination, their identification is possible when they exhibit good delineation and low intensity in T2-weighted images as well as in diffusion-weighted imaging (DWI). Fibroids can also demonstrate atypical features overlapping with those of uterine sarcomas in terms of morphology, signal intensity, and contrast enhancement, making the differential diagnosis challenging [3].

Uterine sarcomas account for 3% to 7% of all uterine cancers, and 1% of all female genital tract cancers [4]. Sarcomas are divided into sarcomas with a cancerous component, constituting 50% of cases, 30% are smooth muscle sarcomas, 20% are endometrial stromal sarcomas (ESSs), and 5% are undifferentiated sarcomas [5,6]. Endometrial stromal tumors are divided into low-grade endometrial stromal sarcoma (LG-ESS), high-grade endometrial stromal sarcoma (HG-ESS), and undifferentiated uterine sarcoma (UUS) [7].

LG-ESS is typically diagnosed in premenopausal women, HG-ESS around the age of 50, and UUS most commonly in the postmenopausal period [8]. LG-ESS has a relatively good prognosis, with a 5-year overall survival (OS) exceeding 90% for stage I, and approximately 50% for stages III and IV. Patients with HG-ESS are typically diagnosed at an advanced stage, with an OS ranging from 11 to 23 months. Similarly to HG-ESS, the expected OS for UUS is also poor, with a duration of less than 2 years [7]. A unifying clinical feature among all these entities is the presence of abnormal uterine bleeding (AUB) [9].

Uterine sarcomas and LMM can present similarly with AUB, which occurs in up to 56% of patients, a palpable pelvic mass present in 54% of patients, and abdominal pain occurring in 22% of patients. Also significant is the similarity in imaging characteristics that complicate preoperative diagnosis [10]. Uterine leiomyosarcoma (LMS) on MRI is characterized by a moderate to high signal intensity on T2-weighted imaging, irregular borders, high signal intensity at high values of DWI, and a decreased apparent diffusion coefficient. The optimal protocol for evaluating the female pelvis in MRI includes axial T1-weighted images in both the phase and out-of-phase, frequency-selective fat-saturated axial T1-weighted images, sagittal images, axial thin-section T2-weighted images, axial DWI, and fat-saturated pre- and post-contrast T1-weighted imaging with subtraction images generated from the post-contrast dataset [11].

Positron emission tomography (PET) [18F] fluorodeoxyglucose (FDG) and PET 16α-[18F]-fluoro-17β-estradiol (FES) imaging are useful tools in the differential diagnosis of uterine smooth muscle tumor and LMS [12]. Uterine sarcomas exhibit greater FDG uptake than LMM, although cases of similar uptake do occur [13]. FDG is associated with glucose-metabolizing cells, so it is not specific for neoplastic transformation [14]. FES PET, on the other hand, provides information on estrogen receptors (ERs) and allows for the exclusion of malignancy in LMM with high FDG uptake. This results from the high presence of ERs in uterine smooth muscle tumors, as confirmed by immunohistochemical studies showing 100% ER expression in LMM [15].

Each of the studies mentioned above presents certain parameters, the differing results of which enable the differential diagnosis of uterine LMM from uterine LMS and endometrial stromal sarcomas. Our aim was to present the diagnostic criteria enabling the differentiation of the discussed tumors. The paper describes three imaging studies: MRI, PET, and US. Each of these studies has been thoroughly described in terms of various parameters, the comprehensive interpretation of which will bring us closer to establishing the correct diagnosis. The differential diagnosis between uterine LMM and LMS is very challenging due to the diversity of these tumors. Therefore, this paper allows for the understanding and comprehension of specific parameters in imaging studies, which will enable the physician interpreting US, MRI, or PET images to make a more confident diagnosis.

## 2. Materials and Methods

The studies included in this review were sourced from databases such as PubMed, Google Scholar, Scopus, and Web of Science. Exclusion criteria included conference abstracts, duplicate papers, incomplete publications without access to source data, and articles written in languages other than English. The quality assessment of the literature was based on the classification of study types according to the EBM (Evidence-Based Medicine) hierarchy. This review is primarily based on sources of moderate to high levels of evidence quality. A qualitative assessment of potential bias was also performed, taking into account study design, inclusion criteria, and outcome reporting. No major methodological concerns were identified, and the overall risk of bias was deemed low to moderate.

Inclusion criteria were as follows: peer-reviewed publications from scientific journals, expert consensus statements, systematic reviews, original studies, cohort studies, retrospective and prospective analyses, and case reports. The final number of articles included was 58. Many of the studies discussed multiple imaging modalities. As a result, the total number of articles primarily or partially addressing MRI was 38, with PET/CT covered in 17 articles, and ultrasound (US) in 19. Among all the articles, 9 were identified as case reports.

## 3. Results

### 3.1. MRI Utility in Differentiation of Specific Uterine Mesenchymal Tumors 

#### 3.1.1. MRI Features of Sarcomas and Fibroids

MRI is the gold standard for distinguishing between uterine fibroids and uterine sarcomas. In the differentiation of sarcomas from fibroids in MRI, the acronym BET1T2ER is useful. In the acronym BET1T2ER, the letter B stands for boundaries. In fibroids these are clear, while in sarcomas they are irregular and nodular. This feature demonstrates a sensitivity of 74–84% and a specificity of 86–91% for LMS [16,17]. The letter E signifies enhancement (with the use of a contrast agent), which in fibroids is varied, including areas without enhancement due to degeneration. In sarcomas, enhancement has an irregular outline, is diverse, and is often accompanied by a lack of enhancement in the central part due to necrosis, yielding a sensitivity of 95–100% and a specificity of 68–73% for LMS. Sarcomas are characterized by an increased early enhancement ratio and higher mean enhancement [16,17]. 

In T1WI SI-T1 weighted imaging, the T1 signal intensity in fibroids is low, while in sarcomas, it is characterized by low signal intensity overall, with high signal intensity observed in regions affected by bleeding. Intralesional hemorrhage is a highly accurate indicator of LMS, demonstrating a sensitivity between 95 and 100% and a specificity ranging from 82 to 95%. In T2WI SI weighted imaging, the T2 signal intensity in fibroids is reduced, whereas in sarcomas, it is heterogeneous and intermediate. Areas of low signal intensity on T2WI may result from flow voids or the presence of intralesional hemosiderin. This characteristic showed a sensitivity of 79–84% and a specificity of 86% for identifying LMS. The letter E denotes endometrial thickening. In fibroids, there is no thickening, while in sarcomas, the thickening is irregular and may take place in up to 35–50% of cases. Regarding R-restricted diffusion, in sarcomas, diffusion is restricted, while in fibroids, diffusion is not limited [16,17]. Table 1 presents a comparison of MRI parameters between sarcoma and benign muscle tumor.

Very often, fibroids have low T2WI SI and low or isointense DWI. On the other hand, in grade 3 fibroids, there is “T2WI-shine through” leading to high DWI. The presence of a significant amount of fluid in fibroids (resulting from the breakdown of smooth muscle) is also significant because it causes an elevated DWI and apparent diffusion coefficient (ADC). Increased values of these parameters indicate the presence of a fibroid with malignant potential, which could lead to a misdiagnosis [18]. Each of the parameters mentioned earlier plays a crucial role in distinguishing between fibroids and sarcomas, but the best among them is DWI. This is due to its high accuracy and 100% sensitivity in detecting restricted diffusion, without false-negative results and with a high negative predictive value (NPV). Unfortunately, evaluating only one parameter shows lower specificity. It is important to be cautious about false restrictions in DWI arising from hemorrhagic areas. In such cases, it is helpful to reference DWI to T2 and T1 images [19].

One of the newer MRI techniques is magnetic resonance spectroscopy (MRS), which, by detecting the levels of peaks of metabolites characteristic of a specific tumor, allows for the identification of a distinctive metabolic profile associated with the tumor. In the case of sarcomas, the levels of choline and lipids are much higher than in the case of uterine fibroids [20]. Conclusions were drawn based on a study that included 14 patients with 21 uterine sarcomas and 51 patients with 84 benign LMMs. A 3-T MRI system was used, including T1- and T2-weighted images as well as DWI. The chi-square test was used to compare the percentage of malignant lesions that showed choline peaks and lipid peaks. A binary classification model utilizing the ADC parameter was applied, with the threshold for distinguishing between benign and malignant lesions determined using the knee point method. The optimal decision threshold was identified as 1.057 [20]. Another technique is magnetic susceptibility-weighted imaging (SWI), which detects hemorrhagic lesions through the detection of their breakdown products, such as deoxyhemoglobin, hemosiderin, and ferritin [21]. The enhanced T2 star-weighted angiography (ESWAN) is a method based on SWI [22]. SWI provides better visualization of the metabolites in hemorrhagic foci in highly malignant brain tumors than T1, allowing for a more accurate assessment. SWI has also been recognized as a valuable method for detecting uterine LMS with hemorrhagic necrosis [23].

Red degeneration, also known as hemorrhagic necrosis, is a hemorrhagic infarction of uterine LMM [24]. The susceptibility-weighted MR sequence (SWS) is a useful method for better differentiating uterine sarcomas with extensive hemorrhagic necrosis from LMM with red degeneration or LMM with other degenerative characteristics. This method increases the sensitivity of MRI compared to blood breakdown products [25]. The researchers Takeuchi et al. showed that SWS is characterized by low intensity at the periphery in every case of LMM with vascular degeneration. SWS enables the detection of hemosiderin deposits indicating the presence of an old venous thrombosis and hemorrhage at various time points. In their paper, they revealed a 100% sensitivity in identifying a peripheral low-intensity rim in all 15 LMMs with red degeneration [25]. T1-weighted fat-saturated images showed high intensity at the periphery in uterine LMM with red degeneration in only 47% of cases, confirming the higher sensitivity of SWS. A similar relationship can be observed in T2-weighted imaging, where only 47% of LMMs with red degeneration showed low signal intensity at the periphery [25].

#### 3.1.2. MRI Features of ESS

On MRI, ESS typically appears as a hypointense lesion on T1-weighted images and a hyperintense and heterogeneous area on T2-weighted images, with strips of hypointense signal representing intact areas of myometrium. Moreover, they show moderate and heterogenous contrast enhancement and high signal with low ADC on DWI [26]. MRI plays a crucial role in the differentiation of ESS subtypes. ESS may mimic the appearance of endometrial polyps or uterine fibroids on imaging. However, unlike fibroids, ESSs typically demonstrate reduced signal intensity on fat-saturated sequences, extensive areas of necrosis, and a distinct low-signal-intensity rim along the periphery on T2-weighted images [27]. A hallmark feature of LG-ESS is the “bag of worms” appearance on T2-weighted sequences, which results from lymphovascular invasion and infiltration into the myometrium. In contrast, HG-ESS is characterized by a peripheral hypointense rim and extensive areas of necrosis [27]. Moreover, HG-ESS typically appears as a large, heterogeneous polypoid mass with frequent myometrial invasion [28]. It often demonstrates hemorrhage, lymphovascular invasion, and necrosis. On contrast-enhanced MRI, it shows heterogeneous and markedly increased enhancement compared to standard myometrium [26,29]. Koichiro Matsuura et al. described a UUS image on MRI as a T2-weighted heterogeneous lesion with an irregular, nodular margin. On T1-weighted sequences it contains hyperintense areas, suggesting hemorrhagic components. Contrast-enhanced T1 images with fat suppression show a feather-like enhancement pattern. Diffusion-weighted imaging reveals marked diffusion restriction, with the lesion exhibiting a stronger signal than the surrounding myometrium and a low ADC value (0.90 × 10^−3^ mm^2^/s), indicative of densely packed tumor cells [30]. Selected MRI features of LG-ESS, HG-ESS, and UUS are presented in the Table 2.

### 3.2. PET Utility in Differentiation of Specific Uterine Mesenchymal Tumors

#### 3.2.1. PET Features of Sarcoma and Fibroids

18F-FDG PET is associated with cellular glucose metabolism, so it is not specific for detecting uterine sarcoma. Uterine fibroids exhibit lower FDG uptake than uterine sarcomas, which corresponds to a lower level of glucose metabolism in the cells. However, there have been cases of increased FDG uptake by uterine fibroids [31]. Fluorodeoxyglucose positron emission tomography/computed tomography (18F-FDG PET/CT) examination allows differentiation between malignant and benign lesions based on functional glucose metabolism, since malignant tissues demonstrate increased glucose metabolism compared to benign tissue. This arises from a greater upregulation of the GLUT-1 transporter in malignant tumors, what further results in increased FDG uptake [32].

FES PET provides information about the activity of ERs, which are significantly more abundant in fibroids than in sarcomas. Therefore, the accumulation of FES was higher in uterine fibroids than in uterine sarcomas. Performing FES PET and 18F-FDG PET examinations allows for better differentiation of the patient’s condition when the 18F-FDG PET results are uncertain [31].

ERs are divided into two types, ERα and ERβ. ERα predominates in uterine fibroids, whereas it is less prevalent in uterine sarcomas and uterine smooth muscle [33]. The expression of the ERβ receptor was the same in uterine sarcoma and uterine fibroids [34]. Therefore, the ERα receptor is the most significant in differentiating the aforementioned conditions. It is important to emphasize that FES PET primarily corresponds to the detection of ERα expression. The 18F-FDG PET/FES PET ratio is also of significant importance, as a higher ratio is observed in malignant tumors. FES PET also serves indirectly to assess progesterone receptors, as there is a good agreement between ERα and the progesterone receptor. Therefore, high expression of progesterone receptors is more prevalent in uterine fibroids than in uterine sarcomas [35]. Table 3 presents differences in 18F-FDG PET and FES PET between uterine fibroids and uterine sarcomas.

The maximum standardized uptake value (SUVmax), a parameter measured using positron emission tomography/computed tomography (PET/CT), enables differentiation between uterine sarcoma and LMM. When it exceeds 7.5, it allows exclusion of LMM with a sensitivity of 73.3%, specificity of 100%, positive predictive value (PPV) of 100%, and NPV of 82.6%. Increasing the diagnostic accuracy is possible by combining PET/CT with LDH, as the sensitivity rate resulting from this combination is 86.6%, with a specificity of 100%, PPV of 100%, and NPV of 90.4% [36].

Cases of increased FDG uptake in PET/CT due to LMM occur in premenopausal women or those with degenerative changes. FDG uptake is also influenced by the menstrual cycle phase and estrogen levels [36,37].

Researchers have also shown that the combination of PET/CT and LDH enables the diagnosis of uterine sarcoma with 100% sensitivity, specificity, accuracy, NPV, and PPV. This particular conclusion was based on the diagnostic evaluation of four cases of LMS [38].

PET or PET/CT scans can also be utilized in diagnosing recurrences of uterine sarcoma, as their sensitivity and specificity were 87.5% and 97.5% for patients without symptoms. However, for symptomatic patients, whose recurrence was confirmed by computed tomography (CT) scan, it was 92.9% and 100% [39].

MRI and PET imaging allow for the differentiation of uterine smooth muscle tumor (SMT) and benign LMM. Lin et al. demonstrated that contrast-enhanced MRI enables the detection of the most characteristic features of uterine smooth muscle tumors, with a higher accuracy than DWI, T2WI, and T1WI [40]. Other researchers, Yoshida et al., found that 18F-FDG PET proved to be a better modality compared to MRI in T1WI and T2WI [13]. Nakagawa et al. suggests that the diagnostic efficacy of PET imaging or a certified radiologist is higher than a single MRI parameter [41]. The study authors suspect hyperintensity in T2WI imaging in atypical smooth muscle tumors and a black-out effect in T2 in typical smooth muscle tumors. Of course, multiparametric MRI imaging demonstrates better efficacy than PET and a certified radiologist [41].

#### 3.2.2. PET Features of ESS

Naohiko Umesaki et al. reported a case of primary ESS detected by 18F-FDG PET, demonstrating an SUV of 5.5. Based on their findings, the authors concluded that 18F-FDG PET can effectively visualize sarcoma lesions, including ESS, and may serve as a valuable diagnostic tool [42]. However, data on the detection of primary ESS using 18F-FDG PET remain limited [43]. Other case-based studies provide additional insight into the role of 18F-FDG PET imaging in ESS assessment. For instance, Gangireddy et al. described an elderly female patient with a recurrent pelvic ESS, in whom 18-FDG PET/CT demonstrated elevated FDG uptake within the mass, suggestive of malignancy and contributing to the diagnosis [44]. Similarly, Fujiishi et al. discussed a case of low-grade ESS in a 69-year-old woman who exhibited an unusually high FDG accumulation on PET imaging. The authors emphasized that although ESS generally shows lower FDG uptake than uterine LMS, the observed metabolic activity in this case aided in distinguishing between these two entities [45].

### 3.3. US Utility in Differentiation of Specific Uterine Mesenchymal Tumors

#### 3.3.1. US Features of Sarcoma and Fibroids

The researchers Russo et al. found that among 70 patients examined with US for centrally richly vascularized myomas, 93% of them had benign tumors, while 7% were found to be malignant on histopathological examination. It is also noteworthy that single lesions in the conducted US examination were more characteristic of adenomas than uterine LMM. The regularity of tumor borders is more characteristic for a LMM than a LMS [46]. The study revealed that 60% of malignant changes had irregular borders. On the other hand, echogenicity was heterogeneous in 72.3% of LMM, and cystic areas occurred in 31.3% of typical smooth muscle tumors, in 55.2% of variants of smooth muscle tumors, and in 40% of malignant changes. When examining eight smooth muscle LMSs, 50% of them had cystic areas. The endometrium was visible in 40% of LMSs on US examination, while it was visible in 81.3% of typical LMMs and in 89.7% of smooth muscle tumor variants [46].

Putra et al. conducted a study on 18 patients with smooth muscle LMS confirmed by histopathological examination. The tumor borders in all patients were irregular, and they became more irregular with tumor progression. Also characteristic was the loss of over 25% of uterine muscle in approximately 61% of patients [47]. Loss of typical benign LMM features was observed in 77.8% of cases, and necrosis was present in 85.7% of cases. Cysts, on the other hand, were present in 83.3%. Using color Doppler, a lack of peripheral vascularity or its reduction was shown in 66.7% of tumors. It was also significant to note the presence of minimal to moderate intratumoral vascularity signals in 85.7% of tumors [47].

Uterine fibroids in US images appear solid, round, and hypoechoic, and exhibit well-defined boundaries. Frequently, fibroids are heterogeneous. Doppler examination allows us to observe the characteristic vascularity of the fibroid at the periphery [48]. Uterine LMSs, in US images, are large and display varied echogenicity. They are characterized by calcifications, absence of shadows, and the presence of irregular cysts [49].

Most commonly, LMSs are richly vascularized in the center of the tumor, although a few rare LMSs are sparsely vascularized or vascularity is not detected [49].

Results of a study by Thangappah et al., however, demonstrated that characteristic features of sarcomas in US, such as diverse echogenicity, the presence of necrosis in the center, and varied tumor vascularity in Doppler imaging, also occur in some fibroids [50]. Table 4 presents the characteristics of LMS and LMM in US examination.

#### 3.3.2. US Features of ESS

On US, ESS typically presents with a nonspecific appearance as a heterogeneous, hypoechoic mass within the endometrium, often showing significant invasion into the myometrium and demonstrating low-resistance blood flow within the lesion on Doppler examination [51]. For instance, Shashank et al. reported a case of a patient with HG-ESS, whose ultrasonography revealed a well-defined, heterogeneous, hypoechoic mass measuring 20 mm × 17 mm within the myometrium of the fundal region, likely representing an intramural fibroid, along with an overall enlarged uterus [52]. Ga Eun Park et al. conducted a retrospective study of US results in 10 patients with confirmed LG-ESS. LG-ESS presented as an intramural mass either extending into the endometrial cavity (*n* = 6) or confined purely within the myometrium (*n* = 4). Lesion size was 6.2 cm on average, varying between 4 and 9.1cm. Imaging characteristics were diverse: most tumors (*n* = 6) were mainly solid with cystic degeneration, one appeared as a mostly unilocular cystic mass, two showed poorly defined infiltrative solid patterns, and one was a well-demarcated solid mass. Of the seven tumors with cystic changes, five displayed multiloculated or clustered cystic areas, while two had simple unilocular cysts [53]. Accurate diagnosis of specific sarcomas can sometimes be challenging, and preoperative histological diagnosis is not always available. Therefore, it is valuable to utilize US as the initial imaging modality and to identify characteristic sonographic features that may aid in further diagnostic evaluation [54]. Ludovisi et al. conducted a retrospective multicenter study, aimed at evaluating the US characteristics of selected uterine sarcomas. The study included, among other investigations, an assessment of 48 cases of ESS. ESS most commonly exhibited a clearly visible normal endometrium (in 44 out of 48) and well-defined tumor margins (in 29 out of 48). ESSs were also less vascularized compared to other types of sarcoma (color score of 1 or 2 in 20 out of 47 cases) in tumors with reliable color-Doppler information. ESSs were often misclassified as benign (in 13 out of 48) [49]. Typical US features are presented in Table 5.

## 4. Discussion

This literature review of the differential diagnosis of malignant mesenchymal tumors has raised many questions: Which imaging studies appear to be the most reliable in differentiating the above-mentioned tumors? Which parameters should we pay the most attention to?

Malignant mesenchymal tumors pose a significant challenge when it comes to differentiation. This is due to the occurrence of similar clinical symptoms in patients and their resemblance in radiological images. This paper describes the parameters of MRI, PET, and US that can help guide the radiologist or gynecologist towards making an accurate diagnosis.

Although MRI is performed with different protocols by different units, clinical results and conclusions are consistent. MRI plays an important role in diagnosis [19]. Although CT is routinely used for the assessment of distant spread, MRI is suggested to be the best modality for assessing uterine sarcomas [16]. Developed by the researchers Smith et al., the acronym BET1T2ER allows for better memorization and systematization of the MRI parameters necessary for differentiating uterine LMM from LMS. The parameters collected in the acronym, such as borders, enhancement, T1WI signal intensity, T2WI signal intensity, endometrial thickening, and restricted diffusion, due to their differences depending on the tumor, guide us towards determining whether we are dealing with an LMM or an LMS [16].

The presence of fluid in a myoma can often mistakenly suggest a uterine LMS on radiological images. Concern in this case is due to the elevated levels of DWI and ADC [18]. There are many different and helpful MRI techniques that detect specific metabolites. For example, the MRS technique detects a characteristic increase in choline and lipid levels typical of LMS [20]. SWI detects hemorrhagic changes based on the breakdown products, useful in the diagnosis of LMS with hemorrhagic necrosis [21,22]. Challenges in differentiating between LMM and LMS also arise when we cannot differentiate hemorrhagic necrosis, characteristic of sarcomas, from red degeneration, more characteristic of LMM. In such cases, the SWS technique appears to be helpful. LMS with vascular degeneration on SWS consistently showed low intensity at the edges [23]. This study, in such situations, can determine the pathology we are dealing with.

A very useful study in differential diagnosis is 18F-FDG PET imaging. Among 18F-FDG PET and FES PET, FES PET appears to be the more reliable study because it correlates with ER alpha, the increased levels of which can be observed in uterine LMM [31]. Analyzing the literature, one can observe better specificity of FES PET for uterine LMM. The 18F-FDG-PET/CT examination goes beyond the current recommendation for the detection of recurrent uterine LMS, which involves periodic physical examination and conventional imaging such as CT or MRI. Its application may be useful in the evaluation of suspected recurrent uterine LMS [32]. PET/CT should also be used in the diagnosis of uterine sarcoma and the differentiation of malignant and benign lesions. A high impact of PET or PET/CT on the post-therapy surveillance of patients with treated uterine sarcoma has been observed [39].

This study also described the 18F-FDG PET/FES PET ratio, and by determining this ratio, it is also possible to differentiate between the discussed tumors, as an increase in this ratio indicates the malignancy of the tumor [35]. The researchers Kusunoki et al., investigating PET/CT associated with LDH, found that it is characterized by a higher sensitivity by 13.3% and an increase in NPV by 7.8% compared to PET/CT in the differential diagnosis. It is also crucial to pay attention to menopause, degenerative changes, menstrual cycle phase, and estrogen levels because these factors can influence the FDG uptake result [36]. Comparing MRI to PET, researchers have differing opinions. Some emphasize the radiologist’s expertise and the greater effectiveness of PET compared to multiparametric MRI. On the other hand, attention is drawn to the higher effectiveness of PET and a certified radiologist compared to a single MRI parameter.

Among all the discussed imaging studies, US appears to be the most accessible and quickest to perform. Therefore, US, being the first-choice method in the diagnosis of endometrial stromal tumors, allows us to determine what we are dealing with.

The researchers Russo et al. conducted a study in which the most common feature indicating malignancy was irregularity of the borders, present in as many as 60% of patients with malignant changes. The least, only 7% of the group of 70 patients with a centrally vascularized suspicion of myoma, were found to have uterine LMS. Putra et al. demonstrated that central vascularity was present in 85.7% of patients out of 18 with histologically confirmed LMS. Central vascularity is a matter of debate, as Russo et al. found a benign tumor in 93% of 70 patients with suspected myoma. Peripheral vascularity is characteristic of LMM, with 66.7% of uterine LMS showing a lack of peripheral vascularity [46,47,48].

ESS on MRI showed heterogeneous isointense areas on T1 images, while hyperintense areas with hypointense margins were observed on T2 images. Also characteristic of ESS on MRI was the presence of polypoid masses of the endometrium. This subtype of the tumor can resemble a polyp or LMS, but it is distinguished by lower enhancement and the extent of necrosis. LG-ESS on MRI presents a characteristic image of a “worm-like” sac on T2, caused by lymphatic and vascular invasion. The images of this subtype vary greatly, characterized by involvement of the uterine myometrium and hypointense bands on T2WI. For HG-ESS, a hypointense margin and extensive necrosis proved to be characteristic [27].

PET has proven to be a more accurate method for assessing response to chemotherapy. The researchers Mehmet Emin Kalender et al. demonstrated that 18F-FDG PET/CT enabled verification of treatment effectiveness by evaluating the SUV parameter, which decreased after chemotherapy. On the other hand, CT did not show any differences in tumor size [55]. PET, used for the diagnosis and differentiation of tumors, has also shown usefulness in treatment monitoring. It allows for the detection of changes caused by chemotherapy that were not assessable with CT. ESS showed more intense FDG PET accumulation than LMM, but less than uterine LMS. LG-ESS exhibited exceptionally high FDG uptake [45,56].

The similarity between tumors such as endometrial stromal sarcoma, uterine LMS, and adenomyosis on US examination poses a challenge for the physician. Of course, there are US features that can guide the physician towards a specific type of tumor, although these tumors often present diagnostic difficulties in differentiation.

In US examination, researchers point to the presence of ESS with diffuse thickening of the uterine muscle, a mass in the central cavity, a mural or polypoid mass, a heterogeneous hypoechoic endometrial mass with extensive involvement of the uterine myometrium, central or peripheral vascularity, low resistance in intratumoral arteries, and a low resistance index [27,54].

Through US examination, we can differentiate LMS from ESS, with features such as a lesion diameter >8 cm indicating ESS, a BMI less than or equal to 20, and a lymphocyte ratio greater than 2.1 [57]. Researchers also found that 91.7% of ESS cases had a visible normal endometrium. 60.4% had regular tumor borders, and 42.6% showed reduced vascularity compared to other sarcomas. On the other hand, USS (uterine smooth muscle tumor) was characterized by 74.2% irregular borders, and 40% showed hemorrhagic or glassy echogenicity of cystic fluid [49]. In the case of LG-ESS, 60% of the tumors invaded the endometrial cavity, 40% were centrally located; 60% had well-differentiated, smooth, and well-defined tumor margins, and 40% had irregular tumor margins; and 70% showed an internally well-defined area of cystic degeneration. LG-ESS tumors had an average maximum tumor diameter of 6.2 cm, and the average size of cysts was 2.8 cm [53]. When examining HG-ESS subtypes through US, noticeable large heterogeneous polypoid masses with distinct vascular invasion were observed. Other researchers, Shashank et al., also demonstrated a well-defined heterogeneous hypoechoic mass measuring 20 mm × 17 mm, with increased vascularity [52,58].

## 5. Conclusions

Analyzing all the above studies, one can conclude that there are parameters in MRI, PET, and US that can guide the physician towards making a diagnosis. This leads to the question of which of these tests is the best. Considering availability and speed of execution, US emerges as the winner. Unfortunately, the characteristics of US for individual tumors may overlap. However, when comparing the effectiveness of methods, it is not possible to unequivocally determine which, MRI or PET, is better, as both methods have parameters with high sensitivity and specificity. An important factor, however, is the skills and experience of the radiologist.

The studies discussed in this article, however, had several limitations. Firstly, these are small patient groups in the studies we used, stemming from the rarity of the tumors we described. Secondly, there is a lack of specific guidelines regarding the criteria for diagnosing malignant uterine mesenchymal tumors through imaging studies.

Therefore, we believe that conducting multiple studies on a larger group of patients is necessary to establish specific guidelines regarding imaging studies that will be most specific to each subtype of the tumor. This will enable radiologists or gynecologists to make a more certain diagnosis and reduce the problem of differentiating these tumors in imaging studies. Due to the rarity of the tumors discussed, achieving a larger patient cohort may prove challenging. A potential solution to this issue could be the standardization of imaging protocols, which would enable comparison and aggregation of results across studies. This is important because it will result in a more precise selection of invasive procedures, without the fear that a LMM will be found to be a malignant sarcoma.

## Figures and Tables

**Table 1:** Comparison of MRI parameters for a sarcoma versus a benign muscle tumor [16].

The Acronym BET1T2ER		
	SARCOMA	FIBROID
B—border	irregular and nodular	clear
E—enhancement	irregular outline	varied
T1—T1WI SI	high signal intensity observed in regions affected by bleeding	low
T2—T2WI SI	heterogeneous and intermediate	reduced
E—Endometrial thickening	the thickening is irregular	no thickening
R—restricted diffusion	restricted	not limited

T1WI SI-T1—weighted imaging T1 signal intensity; T2WI SI T2—weighted imaging T2 signal intensity.

**Table 2:** Selected MRI features of ESS types [26,27,28,29,30].

Type of ESS	Selected MRI Features
LG-ESS	- Appearance of “bag of worms” on T2-weighted sequences
HG-ESS	- Large, heterogeneous polypoid mass with frequent myometrial invasion - Peripheral hypointense rim and extensive areas of necrosis - Heterogeneous and markedly increased enhancement on contrast-enhanced MRI
UUS	- Contains hyperintense areas (suggesting hemorrhagic components) on T1-weighted sequences - Heterogeneous lesion with an irregular, nodular margin on T2-weighted sequences - Feather-like enhancement pattern on contrast-enhanced T1 sequences

LG-ESS—low-grade endometrial stromal sarcoma; HG-ESS—high-grade endometrial stromal sarcoma; UUS—undifferentiated uterine sarcoma.

**Table 3:** Differences in 18F-FDG PET and FES PET between uterine fibroids and uterine sarcomas [31,35].

Type of Tumor	18F-FDG PET	FES PET
**Uterine fibroids**	Typically lower FDG uptake (due to lower level of glucose metabolism in the cells)	Higher FES uptake (due to higher expression of ERα)
**Uterine sarcomas**	Typically higher FDG uptake (due to lower level of glucose metabolism in the cells)	Lower FES uptake (due to lower expression of ERα)

FDG—[18F] Fluorodeoxyglucose; FES—16α-[18F]-fluoro-17β-estradiol; 18F-FDG PET—Fluorodeoxyglucose positron emission tomography; FES PET—Fluoroestradiol positron emission tomography; ERα—Estrogen receptor alpha.

**Table 4:** Features of LMS and leiomyoma on US [46].

LMM Image on US	LMS Image on US
72.3% heterogeneous echogenicity	60% irregular borders
31.3% cystic areas	40–50% cystic areas
81.3% visible endometrium	40% visible endometrium

US—ultrasound; LMS—leiomyosarcoma; LMM—leiomyoma.

**Table 5:** Typical US features of ESS [49,51].

Typical US Features of ESS:
- Nonspecific appearance as heterogeneous, hypoechoic mass within endometrium - Often show invasion into the myometrium - Demonstrate low-resistance blood flow within the lesion - Clearly visible normal endometrium - Well-defined tumor margins

ESS—endometrial stromal sarcoma; US—ultrasound.

## Data Availability

The data used in this article were sourced from the materials mentioned in the References section.

## Abbreviations

The following abbreviations are used in this manuscript:

AUB	Abnormal uterine bleeding
EBM	Evidence-based medicine
US	Ultrasound
MRI	Magnetic resonance imaging
PET	Positron emission tomography
DWI	Diffusion-weighted imaging
FDG	[18F] Fluorodeoxyglucose
FES	16α-[18F]-fluoro-17β-estradiol
ER	Estrogen receptor
LG-ESS	Low-grade endometrial stromal sarcoma
HG-ESS	High-grade endometrial stromal sarcoma
UUS	Undifferentiated uterine sarcoma
LMM	Leiomyoma
LMS	Leiomyosarcoma
OS	Overall survival
T1WI SI-T1	Weighted imaging T1 signal intensity
T2WI SI-T2	Weighted imaging T2 signal intensity
ADC	Apparent diffusion coefficient
NPV	Negative predictive value
MRS	Magnetic resonance spectroscopy
SWI	Susceptibility-weighted imaging
ESWAN	Enhanced T2 star-weighted angiography
SWS	Susceptibility-weighted MR sequence
PPV	Positive predictive value
CT	Computed tomography
SMT	Smooth muscle tumor
PET/CT	Positron emission tomography/computed tomography
18F-FDG PET	Fluorodeoxyglucose positron emission tomography
18F-FDG PET/CT	Fluorodeoxyglucose positron emission tomography/computed tomography
FES PET	Fluoroestradiol positron emission tomography
FES-PET/CT	Fluorostradiol positron emission tomography/computed tomography
ESS	Endometrial stromal sarcomas

## References

[1] Seidman J.D., Thomas R.M. (1993). Multiple plexiform tumorlets of the uterus. Arch. Pathol. Lab. Med..

[2] Khan A.T., Shehmar M., Gupta J.K. (2014). Uterine fibroids: Current perspectives. Int. J. Womens Health.

[3] Méndez R.J. (2020). MRI to Differentiate Atypical Leiomyoma from Uterine Sarcoma. Radiology.

[4] Major F.J., Blessing J.A., Silverberg S.G., Morrow C.P., Creasman W.T., Currie J.L., Yordan E., Brady M.F. (1993). Prognostic factors in early-stage uterine sarcoma: A gynecologic oncology group study. Cancer.

[5] Mbatani N., Olawaiye A.B., Prat J. (2018). Uterine sarcomas. Int. J. Gynecol. Obstet..

[6] Amant F., Floquet A., Friedlander M., Kristensen G., Mahner S., Nam E.J., Powell M.A., Ray-Coquard I., Frcog N.S., Sykes P. (2014). Gynecologic Cancer InterGroup (GCIG) Consensus Review for Endometrial Stromal Sarcoma. Int. J. Gynecol. Cancer.

[7] Horng H.C., Wen K.C., Wang P.H., Chen Y.-J., Yen M.-S., Ng H.-T., Chang Y.-H., Chang Y., Chao H.-T., Chao K.-C. (2016). Uterine sarcoma Part II—Uterine endometrial stromal sarcoma: The TAG systematic review. Taiwan. J. Obstet. Gynecol..

[8] Liu J., Wang Z. (2022). Advances in the Preoperative Identification of Uterine Sarcoma. Cancers.

[9] Capozzi V.A., Monfardini L., Ceni V., Cianciolo A., Butera D., Gaiano M., Berretta R. (2020). Endometrial stromal sarcoma: A review of rare mesenchymal uterine neoplasm. J. Obstet. Gynaecol. Res..

[10] Perri T., Korach J., Sadetzki S., Oberman B., Fridman E., Ben-Baruch G. (2009). Uterine Leiomyosarcoma: Does the Primary Surgical Procedure Matter?. Int. J. Gynecol. Cancer.

[11] Hindman N., Kang S., Fournier L., Lakhman Y., Nougaret S., Reinhold C., Sadowski E., Huang J.Q., Ascher S. (2023). MRI Evaluation of Uterine Masses for Risk of Leiomyosarcoma: A Consensus Statement. Radiology.

[12] Tsujikawa T., Yoshida Y., Mori T., Kurokawa T., Fujibayashi Y., Kotsuji F., Okazawa H. (2008). Uterine Tumors: Pathophysiologic Imaging with 16α-[18F]fluoro-17β-estradiol and 18F Fluorodeoxyglucose PET—Initial Experience. Radiology.

[13] Yoshida Y., Kurokawa T., Sawamura Y., Shinagawa A., Tsujikawa T., Okazawa H., Tsuchida T., Imamura Y., Suganuma N., Kotsuji F. (2008). Comparison of 18F-FDG PET and MRI in Assessment of Uterine Smooth Muscle Tumors. J. Nucl. Med..

[14] Rohren E.M., Turkington T.G., Coleman R.E. (2004). Clinical Applications of PET in Oncology. Radiology.

[15] Bodner K., Bodner-Adler B., Kimberger O., Czerwenka K., Leodolter S., Mayerhofer K. (2003). Estrogen and progesterone receptor expression in patients with uterine leiomyosarcoma and correlation with different clinicopathological parameters. Anticancer. Res..

[16] Smith J., Zawaideh J.P., Sahin H., Freeman S., Bolton H., Addley H.C. (2021). Differentiating uterine sarcoma from leiomyoma: BET1T2ER Check!. Br. J. Radiol..

[17] Lakhman Y., Veeraraghavan H., Chaim J., Feier D., Goldman D.A., Moskowitz C.S., Nougaret S., Sosa R.E., Vargas H.A., Soslow R.A. (2017). Differentiation of Uterine Leiomyosarcoma from Atypical Leiomyoma: Diagnostic Accuracy of Qualitative MR Imaging Features and Feasibility of Texture Analysis. Eur. Radiol..

[18] Suzuki Y., Wada S., Nakajima A., Fukushi Y., Hayashi M., Matsuda T., Asano R., Sakurai Y., Noguchi H., Shinohara T. (2018). Magnetic Resonance Imaging Grading System for Preoperative Diagnosis of Leiomyomas and Uterine Smooth Muscle Tumors. J. Minim. Invasive Gynecol..

[19] Rosa F., Martinetti C., Magnaldi S., Rizzo S., Manganaro L., Migone S., Ardoino S., Schettini D., Marchiolè P., Ragusa T. (2023). Uterine mesenchymal tumors: Development and preliminary results of a magnetic resonance imaging (MRI) diagnostic algorithm. Radiol. Med..

[20] Rahimifar P., Hashemi H., Malek M., Ebrahimi S., Tabibian E., Alidoosti A., Mousavi A., Yarandi F. (2019). Diagnostic value of 3 T MR spectroscopy, diffusion-weighted MRI, and apparent diffusion coefficient value for distinguishing benign from malignant myometrial tumours. Clin. Radiol..

[21] Ulas S.T., Diekhoff T., Hermann K.G.A., Poddubnyy D., Hamm B., Makowski M.R. (2019). Susceptibility-weighted MR imaging to improve the specificity of erosion detection: A prospective feasibility study in hand arthritis. Skeletal Radiol..

[22] Han X., Sun M., Wang M., Fan R., Chen D., Xie L., Liu A. (2019). The enhanced T2 star weighted angiography (ESWAN) value for differentiating borderline from malignant epithelial ovarian tumors. Eur. J. Radiol..

[23] Takeuchi M., Matsuzaki K., Harada M. (2019). Clinical utility of susceptibility-weighted MR sequence for the evaluation of uterine sarcomas. Clin. Imaging.

[24] Kawakam S., Togashi K., Konishi I., Kimura I., Fukuoka M., Mori T., Konishi J. (1994). Red degeneration of uterine leiomyoma: MR appearance. J. Comput. Assist. Tomogr..

[25] Takeuchi M., Matsuzaki K., Bando Y., Harada M. (2019). Evaluation of Red Degeneration of Uterine Leiomyoma with Susceptibility-weighted MR Imaging. Magn. Reson. Med. Sci. MRMS Off. J. Jpn. Soc. Magn. Reson. Med..

[26] Santos P., Cunha T.M. (2015). Uterine sarcomas: Clinical presentation and MRI features. Diagn. Interv. Radiol..

[27] Adiga C.P., Gyanchandani M., Goolahally L.N., Itagi R.M., Kalenahalli K.V. (2016). Endometrial stromal sarcoma: An aggressive uterine malignancy. J. Radiol. Case Rep..

[28] Rha S.E., Byun J.Y., Jung S.E., Lee S.L., Cho S.M., Hwang S.S., Lee HGet a.l. (2003). CT and MRI of Uterine Sarcomas and Their Mimickers. AJR Am. J. Roentgenol..

[29] Toprak U., Paşaoğlu E., Karademir M.A., Gülbay M. (2004). Sonographic, CT, and MRI findings of endometrial stromal sarcoma located in the myometrium and associated with peritoneal inclusion cyst. AJR Am. J. Roentgenol..

[30] Matsuura K., Inoue K., Hoshino E., Yasuda M., Hasegawa K., Okada Y., Baba Y., Kozawa E. (2022). Utility of magnetic resonance imaging for differentiating malignant mesenchymal tumors of the uterus from T2-weighted hyperintense leiomyomas. Jpn. J. Radiol..

[31] Yoshida Y., Kiyono Y., Tsujikawa T., Kurokawa T., Okazawa H., Kotsuji F. (2011). Additional value of 16α-[18F]fluoro-17β-oestradiol PET for differential diagnosis between uterine sarcoma and leiomyoma in patients with positive or equivocal findings on [18F]fluorodeoxyglucose PET. Eur. J. Nucl. Med. Mol. Imaging.

[32] Kao Y.H., Saad U., Tan A.E., Magsombol B.M., Padhy A.K. (2011). Fluorine-18-fluorodeoxyglucose PET/CT for the evaluation of suspected recurrent uterine leiomyosarcomas. Acta Radiol..

[33] Wang H., Wu X., Englund K., Masironi B., Eriksson H., Sahlin L. (2001). Different expression of estrogen receptors alpha and beta in human myometrium and leiomyoma during the proliferative phase of the menstrual cycle and after GnRHa treatment. Gynecol. Endocrinol. Off. J. Int. Soc. Gynecol. Endocrinol..

[34] Huang G.S., Arend R.C., Li M., Gunter M.J., Chiu L.G., Horwitz S.B., Goldberg G.L. (2009). Tissue microarray analysis of hormonal signaling pathways in uterine carcinosarcoma. Am. J. Obstet. Gynecol..

[35] Zhao Z., Yoshida Y., Kurokawa T., Kiyono Y., Mori T., Okazawa H. (2013). 18 F-FES and 18 F-FDG PET for Differential Diagnosis and Quantitative Evaluation of Mesenchymal Uterine Tumors: Correlation with Immunohistochemical Analysis. J. Nucl. Med..

[36] Kusunoki S., Terao Y., Ujihira T., Fujino K., Kaneda H., Kimura M., Ota T., Takeda S. (2017). Efficacy of PET/CT to exclude leiomyoma in patients with lesions suspicious for uterine sarcoma on MRI. Taiwan. J. Obstet. Gynecol..

[37] Tsuchida T., Okazawa H., Mori T., Kobayashi M., Yoshida Y., Fujibayashi Y., Itoh H. (2007). In vivo imaging of estrogen receptor concentration in the endometrium and myometrium using FES PET—Influence of menstrual cycle and endogenous estrogen level. Nucl. Med. Biol..

[38] Nagamatsu A., Umesaki N., Li L., Tanaka T. (2010). Use of 18F-fluorodeoxyglucose positron emission tomography for diagnosis of uterine sarcomas. Oncol. Rep..

[39] Park J.-Y., Kim E.N., Kim D.-Y., Suh D.-S., Kim J.-H., Kim Y.-M., Kim Y.-T., Nam J.-H. (2008). Role of PET or PET/CT in the post-therapy surveillance of uterine sarcoma. Gynecol. Oncol..

[40] Lin G., Yang L., Huang Y., Ng K., Ng S., Ueng S., Chao A., Yen T., Chang T., Lai C. (2016). Comparison of the diagnostic accuracy of contrast-enhanced MRI and diffusion-weighted MRI in the differentiation between uterine leiomyosarcoma/smooth muscle tumor with uncertain malignant potential and benign leiomyoma. J. Magn. Reson. Imaging.

[41] Nakagawa M., Nakaura T., Namimoto T., Iyama Y., Kidoh M., Hirata K., Nagayama Y., Oda S., Sakamoto F., Shiraishi S. (2019). A multiparametric MRI-based machine learning to distinguish between uterine sarcoma and benign leiomyoma: Comparison with 18F-FDG PET/CT. Clin. Radiol..

[42] Umesaki N., Tanaka T., Miyama M., Kawamura N., Ogita S., Kawabe J., Okamura T., Koyama K., Ochi H. (2001). Positron emission tomography with (18)F-fluorodeoxyglucose of uterine sarcoma: A comparison with magnetic resonance imaging and power Doppler imaging. Gynecol. Oncol..

[43] Inoue K., Kuroshima M., Murata Y., Morita H. (2024). A Rare Case of Low-Grade Endometrial Stromal Sarcoma Invading an Old Leiomyoma. Cureus.

[44] Gangireddy M., Chan Gomez J., Kanderi T., Joseph M., Kundoor V. (2020). Recurrence of Endometrial Stromal Sarcoma, Two Decades Post-Treatment. Cureus.

[45] Fujiishi K., Nagata S., Kano R., Kubo C., Shirayanagi M., Ozaki M., Yamamoto T., Nakanishi K., Kamiura S., Nakatsuka S.-I. (2019). JAZF1-SUZ12 endometrial stromal sarcoma forming subserosal masses with extraordinary uptake of fluorodeoxyglucose on positron emission tomography: A case report. Diagn. Pathol..

[46] Russo C., Camilli S., Martire F.G., Di Giovanni A., Lazzeri L., Malzoni M., Zupi E., Exacoustos C. (2022). Ultrasound features of highly vascularized uterine myomas (uterine smooth muscle tumors) and correlation with histopathology. Ultrasound Obstet. Gynecol..

[47] Putra A.D., Maharani N., Gianina K. (2022). Ultrasound Features and Diagnostic Workup of Uterine Leiomyosarcomas. J. Ultrasound Med..

[48] Palheta M.S., Medeiros Fdas C., Severiano A.R.G. (2023). Reporting of uterine fibroids on ultrasound examinations: An illustrated report template focused on surgical planning. Radiol. Bras..

[49] Ludovisi M., Moro F., Pasciuto T., Di Noi S., Giunchi S., Savelli L., Pascual M.A., Sladkevicius P., Alcazar J.L., Franchi D. (2019). Imaging in gynecological disease (15): Clinical and ultrasound characteristics of uterine sarcoma. Ultrasound Obstet. Gynecol..

[50] Thangappah R.B.P. (2019). Uterine Sarcoma: A Clinico-Pathological Study. J. Obstet. Gynecol. India.

[51] Gandolfo N., Gandolfo N.G., Serafini G., Martinoli C. (2000). Endometrial stromal sarcoma of the uterus: MR and US findings. Eur. Radiol..

[52] Shekhar S., Sharma C., Elhence P., Bansal S., Garg N. (2019). A Case of High-grade Endometrial Stromal Sarcoma: A Poignant Allegory. J.-Life Health.

[53] Park G.E., Rha S.E., Oh S.N., Lee A., Lee K.H., Kim M.R. (2016). Ultrasonographic findings of low-grade endometrial stromal sarcoma of the uterus with a focus on cystic degeneration. Ultrasonography.

[54] Oh J., Park S.B., Park H.J., Lee E.S. (2019). Ultrasound Features of Uterine Sarcomas. Ultrasound Q..

[55] Kalender M.E., Sevinc A., Yilmaz M., Ozsarac C., Camci C. (2009). Detection of complete response to imatinib mesylate (Glivec^®^/Gleevec^®^) with 18F-FDG PET/CT for low-grade endometrial stromal sarcoma. Cancer Chemother. Pharmacol..

[56] Maruyama S., Sato Y., Satake Y., Mise H., Kim T. (2015). Diffusion-Weighted MRI and FDG-PET in Diagnosis of Endometrial Stromal Nodule. Case Rep. Obstet. Gynecol..

[57] Cotzia P., Benayed R., Mullaney K., Oliva E., Felix A., Ferreira J., Soslow R.A., Antonescu C.R., Ladanyi M., Chiang S. (2019). Undifferentiated Uterine Sarcomas Represent Under-Recognized High-grade Endometrial Stromal Sarcomas. Am. J. Surg. Pathol..

[58] Tirumani S.H., Ojili V., Shanbhogue A.K.P., Fasih N., Ryan J.G., Reinhold C. (2013). Current concepts in the imaging of uterine sarcoma. Abdom. Imaging.

